# Comparing socioeconomic inequalities between early neonatal mortality and facility delivery: Cross-sectional data from 72 low- and middle-income countries

**DOI:** 10.1038/s41598-019-45148-5

**Published:** 2019-07-05

**Authors:** Terhi J. Lohela, Robin C. Nesbitt, Juha Pekkanen, Sabine Gabrysch

**Affiliations:** 1 Department of Public Health, University of Helsinki, Helsinki, Finland; 2 Heidelberg Institute of Global Health, Heidelberg University, Heidelberg, Germany; 3 Environmental Health Unit, National Institute for Health and Welfare, Helsinki, Finland; 4 Research Department 2, Potsdam Institute for Climate Impact Research, Potsdam, Germany; 5 Institute of Public Health, Charité - Universitätsmedizin Berlin, Berlin, Germany; 6 Faculty of Epidemiology and Population Health, London School of Hygiene & Tropical Medicine, London, UK

**Keywords:** Epidemiology, Epidemiology

## Abstract

Facility delivery should reduce early neonatal mortality. We used the Slope Index of Inequality and logistic regression to quantify absolute and relative socioeconomic inequalities in early neonatal mortality (0 to 6 days) and facility delivery among 679,818 live births from 72 countries with Demographic and Health Surveys. The inequalities in early neonatal mortality were compared with inequalities in postneonatal infant mortality (28 days to 1 year), which is not related to childbirth. Newborns of the richest mothers had a small survival advantage over the poorest in unadjusted analyses (−2.9 deaths/1,000; OR 0.86) and the most educated had a small survival advantage over the least educated (−3.9 deaths/1,000; OR 0.77), while inequalities in postneonatal infant mortality were more than double that in absolute terms. The proportion of births in health facilities was an absolute 43% higher among the richest and 37% higher among the most educated compared to the poorest and least educated mothers. A higher proportion of facility delivery in the sampling cluster (e.g. village) was only associated with a small  decrease in early neonatal mortality. In conclusion, while socioeconomically advantaged mothers had much higher use of a health facility at birth, this did not appear to convey a comparable survival advantage.

## Introduction

Every year, nearly two million babies die during their first week of life, accounting for over a third of global under-five mortality^1^. Over the past decades, mortality during the early neonatal period (0 to 6 days) declined more slowly than late neonatal (7 to 27 days) and postneonatal infant (28 days to 1 year) mortality^2^. The majority of early neonatal deaths occur in low- and middle-income countries and the main medical causes are prematurity and intrapartum-related events, such as birth asphyxia, whereas infections are responsible for most late neonatal and infant deaths^2–6^. Consequently, early neonatal survival is closely related to care at birth, whereas postneonatal infant survival depends rather on good care practices at home (including feeding and hygiene), and on care-seeking for sickness.

**Figure 1:**
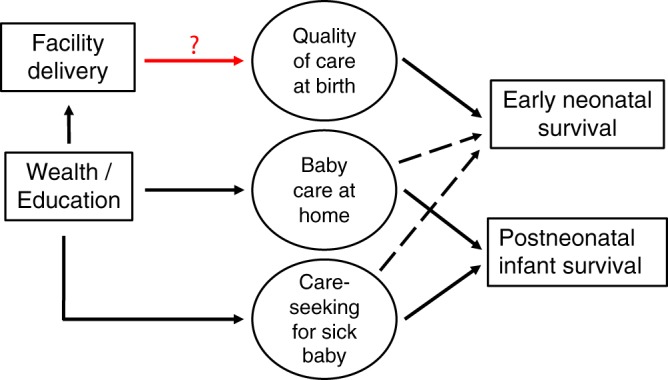
Pathways through which household wealth and maternal education could increase early neonatal and postneonatal infant survival. Higher wealth and education increase facility delivery and are thought to improve postneonatal survival mainly through good care practices at home and through timely care-seeking for the baby. Facility delivery can, but does not automatically ensure, high quality of care (red arrow) and any early neonatal survival benefit conferred by facility delivery depends largely on quality of care at birth. Pathways postulated to be stronger are represented by continuous arrows and weaker pathways by dashed arrows. Measured factors are represented squared while unmeasured factors are circled.

Skilled attendance at birth is the main intervention through which early neonatal lives could be saved and, in most contexts, requires care in a health facility^7,8^. Recent years have seen a substantial increase in facility deliveries, with three out of four deliveries now happening inside health facilities, but not an equivalent reduction in early neonatal mortality^9^. It has been suggested that this mismatch is due to low quality of care at birth in many countries^8^.

An analysis of DHS data from 67 low-and middle-income countries found only weak evidence for an individual-level association between facility delivery and early neonatal mortality^10^. Studying the influence of facility delivery on mortality directly can be misleading, especially in settings where women deliver at home as long as all is well and seek care only when complications arise. Comparing mortality between home births and facility births then leads to an unfair comparison as health facilities manage the complicated cases with a higher risk of death^11^. In this paper, we refer to this adverse selection of high-risk deliveries into health facilities as “confounding by case-mix”.

An indirect approach to studying the influence of facility delivery on mortality is to investigate facility delivery at the country or community level instead of at the individual level^11^, as with larger numbers, the average risk of complications per unit of analysis will be more similar. A limitation of country-level correlation analyses is that they can be confounded by country-level factors, such as factors related to health systems^11^. Therefore, community-level analyses have been used in previous studies to avoid confounding by case-mix inherent to individual-level analyses^12,13^.

Another indirect way to explore the link between facility delivery and early neonatal survival is to study socioeconomic inequalities. Higher socioeconomic status is a distal determinant that acts through various proximal factors to improve early neonatal survival, including care at birth (Fig. 1)^14^. In high-mortality settings with large socioeconomic inequalities in facility delivery, we should expect large inequalities in early neonatal mortality - provided that facilities save lives through high-quality delivery care^15,16^. If, however, no high-quality care at birth is available in a country, socioeconomic advantage in care-seeking cannot translate into better survival, and early neonatal mortality would thus be high among all socioeconomic groups.

Socioeconomic inequalities in under-five mortality are large and well documented^2,17–22^. However, similar large-scale evidence does not exist for early neonatal mortality. Indeed, there are only two multi-country analyses from low- and middle-income countries, including six^23^ and seven^24^ countries with sub-national data, and some sub-national or facility-based studies reporting socioeconomic inequalities in early neonatal mortality. Further, no previous study has compared socioeconomic inequalities in early neonatal mortality with coverage of facility delivery on a large scale.

In this paper, we aim to assess to what extent socioeconomic inequalities in facility delivery correspond to socioeconomic inequalities in early neonatal survival. First, we describe socioeconomic inequalities in early neonatal mortality in 72 countries in relation to average early neonatal mortality in each country. We then quantify the associations of higher household wealth and higher maternal education with risk of early neonatal death (i.e. mortality strongly related to childbirth), and for comparison with postneonatal infant death (i.e. mortality mainly unrelated to childbirth). Finally, we quantify the effects of wealth and education on facility delivery and the effect of facility delivery at the cluster level on early neonatal mortality, adjusting for wealth, education and residence.

## Methods

### Ethics

We used publicly available data collected by the Demographic and Health Survey (DHS) program. The ethical issues and consent procedures have been described in detail (http://www.dhsprogram.com).

### Data sources

We analyzed cross-sectional DHS data collected after 1990 from all 72 countries with standard children’s datasets that included information on household wealth and mother’s years of education. The majority of datasets (64 out of 72) were collected in 2000 or later. We used one survey per country, using the latest dataset available by December 13^th^ 2017 in all countries, except for Bangladesh, Colombia and Peru where we used a prior survey as there was substantial missing information on delivery location in the most recent dataset.

The surveys were conducted between 1990 and 2016 and include countries from all six regions defined by the World Health Organization: 37 countries in the African region, 11 in the Americas, ten in Europe, six in South-East Asia, five in the Eastern Mediterranean and three in the Western Pacific. The surveys employ a two-stage random sampling design, and collect date of birth and age at death of all children born to women of reproductive age (15 to 49 years) in the five years prior to the survey, maternal education, household wealth and residence. DHS methodology is described in detail elsewhere (http://www.dhsprogram.com).

### Measures

Early neonatal death is defined as the death of a live-born baby during the first week of life (0 to 6 days). Postneonatal infant death is defined as the death of an infant after the neonatal period and before one year of age (day 28 to 1 year). As measures of socioeconomic status, we used mother’s years of education and the DHS wealth index which is derived through principal component analysis of household assets^25^.

### Statistical analyses

We calculated the differences in early neonatal mortality, in postneonatal infant mortality and in facility delivery between newborns of the richest and most educated mothers compared to the poorest and least educated mothers, respectively. We estimated absolute inequality with the slope index of inequality (SII) and relative inequality with the relative index of inequality (RII) using logistic regression. The SII and RII are recommended for use in studies on socioeconomic health inequalities as this approach takes into account the entire wealth and education distributions^26^.

The SII was created by assigning each child a continuous rank between zero and one based on their household wealth or mother’s education within each country^27,28^. The rank is defined as the proportion of the population with a lower position in the hierarchy. The richest person has thus rank 1 and the poorest rank 0. The associations between the wealth and education rank variables and the three outcomes (early neonatal mortality, postneonatal infant mortality and facility delivery) were analyzed separately for each country. Wealth rank (for analyses of education), education rank (for analyses of wealth) and place of residence (urban, rural; for both analyses) were included as covariates in adjusted multivariable logistic regression analyses to examine the inequality in the two mortality outcomes and in facility delivery due to wealth and education. Marginal probabilities were used to estimate absolute differences in risk of death between the extremes of the wealth and education ranks (presented as deaths per 1,000 live births (SII)).

Odds Ratios are presented as a relative measure of inequality (RII). The odds ratios (i.e. the RII) and the absolute differences in mortality (i.e. the SII) that we report in this study thus refer to differences across the entire wealth and education distributions. We defined wealth- and education-related inequalities of 0 ±5 per 1,000 live births as ‘small’. This definition was made *a priori* to analysing the data and based on mortality rates in high-income countries that are typically less than 5 per 1,000 live births and generally considered to be low^9^.

We excluded 380 out of 680,198 (0.06%) births from 42 countries from all analyses due to missing information on age at death or mother’s years of education. For the analyses on facility delivery, twins and triplets were counted as one delivery reducing the sample size by 9,711 to 670,107 deliveries. Due to missing information on delivery location, 2,629 out of the remaining 670,107 deliveries (0.4%) were excluded from the analyses of facility delivery.

To investigate the survival benefit associated with facility delivery, we studied associations between average proportion of facility delivery in the sampling cluster (typically referring to a community, for example a village or suburb) and early neonatal mortality using the SII and RII. This approach is based on the assumption that the average proportion of complicated deliveries (i.e. the case-mix) is similar across clusters. Clusters were assigned a rank based on the average proportion of facility delivery in that cluster. We used multivariable logistic regression adjusting for place of residence, average wealth rank and average education rank in the cluster. No evidence of collinearity between cluster-level wealth or education and proportion of facility delivery in the sampling cluster was found based on variance inflation factors and Spearman correlation.

We accounted for the stratified and clustered survey design of the DHS by using sample weights and robust standard errors. To account for heterogeneity in early neonatal mortality related to socioeconomic inequalities between countries, estimates from all countries were pooled using random-effects meta-analyses with the inverse-variance DerSimonian and Laird method. All analyses were conducted using Stata/MP2 15.1 with the svy suite of survey data commands.

### Sensitivity analyses

In addition to using ranks for absolute and relative inequalities in early neonatal mortality, we calculated the mortality difference between the highest and lowest household wealth quintiles (as defined by the DHS) and between mothers with higher education level and those with no education.

As another sensitivity analysis, we used skilled birth attendant (as defined in the DHS country reports) as an outcome instead of facility delivery. Out of the 666,823 deliveries for which information on place of delivery and birth attendant was available, 98% were assisted by a skilled attendant and 93% of deliveries that were attended by a skilled birth attendant took place in a health facility. Twins and triplets were counted as one delivery in this analysis. Information on delivery attendant was missing in 2,693 of 670,107 deliveries (0.4%), and these were excluded from analyses on skilled birth attendants.

## Results

The unweighted study population comprised 679,818 children and 667,478 deliveries for which information on place of delivery was available. There were 13,643 early neonatal deaths (weighted pooled mortality of 18.5 [95% CI: 16.8, 20.2] per 1,000 live births) and 12,920 postneonatal infant deaths (weighted pooled mortality of 17.1 [95% CI: 15.1, 19.1] per 1,000 live births). The weighted pooled prevalence of facility delivery was 68% (95% CI: 64, 72).

### Socioeconomic inequalities in early neonatal mortality

**Figure 2:**
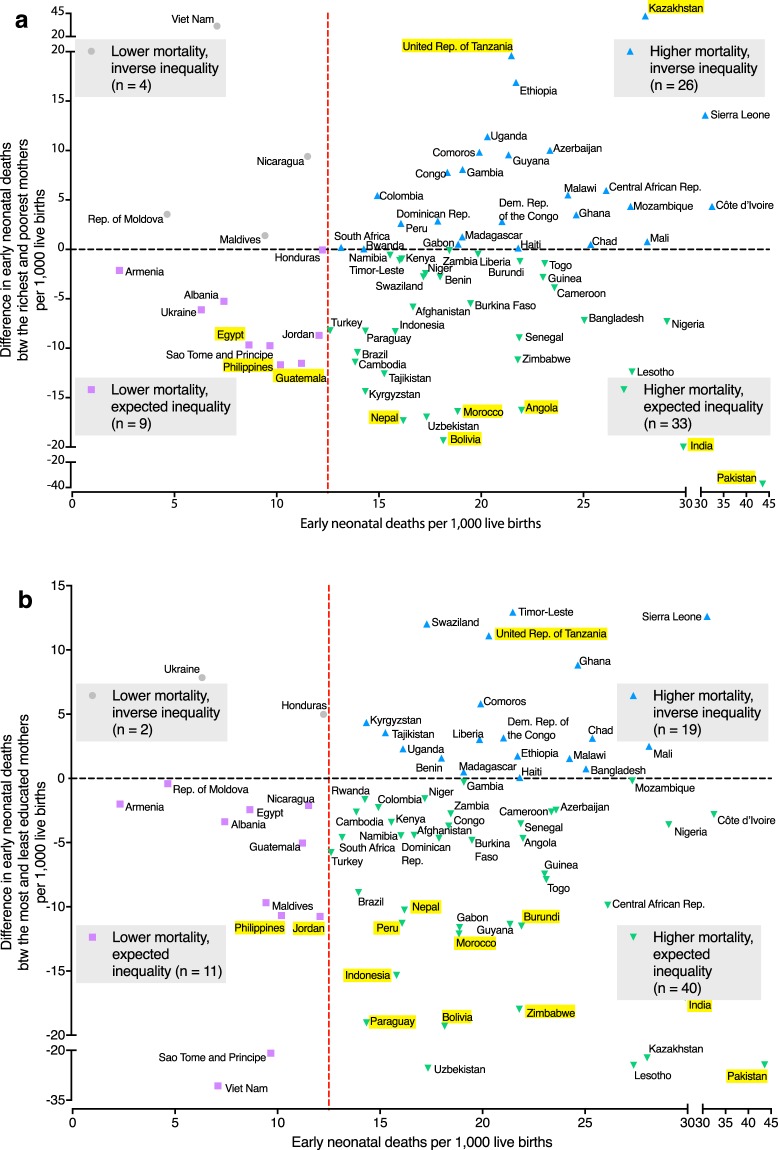
Average early neonatal mortality and unadjusted wealth-related (**a**) and education-related (**b**) inequalities in early neonatal mortality in 72 low- and middle-income Demographic and Health Survey countries. The graphs show a reference line for the Sustainable Development Goal mortality target of 12 neonatal deaths per 1,000 live births. In countries above the zero line for inequality, mortality is *higher* among the wealthier or the more educated, i.e. inverse to what one would expect. Countries with significant inequalities (p < 0.05) are highlighted in yellow. Sample weights and robust standard errors were used in analyses.

We plotted average early neonatal mortality against inequalities in mortality related to household wealth (a) and maternal education (b) (Fig. 2). Early neonatal mortality (i.e. death within a week of birth) exceeded the Sustainable Development Goal (SDG) target for overall neonatal deaths (i.e. within 28 days) of 12 per 1,000 births in 59 out of 72 countries (82%), and we defined mortality as high in these countries.

In 42 out of 72 (58%) countries, the richest had a survival advantage over the poorest, but these inequalities reached statistical significance (p < 0.05) in only nine countries (13%). The poorest survived better compared with the richest in 30 countries (42%) although these reverse inequalities were significant in two countries (3%) only. Newborns of the most educated survived better compared with the least educated mothers in 51 countries (71%) and inequalities reached statistical significance in 12 countries (17%). The less educated had better survival than the more educated in 21 countries (29%), and these reverse education-related inequalities were significant in one country (1%).

The wealth-related inequalities were small (i.e. 0 ± 5) in 29 (40%) countries and the education-related inequalities were small in 39 (54%) countries. Country-level estimates with confidence intervals are presented in Supplementary Table S1.

### Comparison of inequalities in early neonatal mortality and postneonatal infant mortality

Country-level comparisons show that socioeconomic inequalities were generally much smaller for early neonatal than for postneonatal infant mortality (Supplementary Fig. S1). In unadjusted analysis, early neonatal mortality was estimated at 19.9 deaths per 1,000 live births among the poorest and at 17.0 deaths per 1,000 live births among the richest translating into an inequality of 2.9 deaths per 1,000 live births (Table 1). The unadjusted education-related inequality was 3.9 deaths per 1,000 live births, as there were 20.3 deaths per 1,000 live births among newborns of the least educated and 16.4 deaths per 1,000 live births among the most educated mothers. Adjusting for education weakened the wealth-related inequality to 1.8 deaths per 1,000 live births, and adjusting for wealth and residence weakened the education-related inequality to 2.5 per 1,000 live births (Table 1). These inequalities are considerably smaller than the pooled inequalities in postneonatal infant mortality (Table 1). Using household wealth quintiles and educational level instead of using ranks led to similar results (Supplementary Fig. S2).

**Table 1:** Individual-level wealth- and education-related inequalities in early neonatal and postneonatal infant mortality. Pooled unadjusted and adjusted estimates for 72 low- and middle-income Demographic and Health Survey countries. N = 679,818 live births.

Wealth-related inequalities Richest *versus* poorest	Early neonatal mortality		Postneonatal infant mortality	
	Difference in mortality per 1,000 live births (95% CI)	Odds Ratio of mortality (95% CI)	Difference in mortality per 1,000 live births (95% CI)	Odds Ratio of mortality (95% CI)
Unadjusted	−2.9 (−5.0, −0.8)	0.86 (0.76, 0.97)	−10.4 (−12.8, −8.0)	0.50 (0.42, 0.59)
Adjusted for residence^a^	−2.9 (−4.9, −0.9)	0.87 (0.77, 0.98)	−9.7 (−12.3, −7.1)	0.51 (0.43, 0.61)
Adjusted for education rank	−1.8 (−3.7, 0.0)	0.92 (0.81, 1.03)	−7.1 (−9.2, −4.9)	0.62 (0.53, 0.72)
Adjusted for both covariates^b^	−1.8 (−3.6, −0.1)	0.93 (0.82, 1.04)	−6.4 (−8.7, −4.2)	0.64 (0.55, 0.76)
**Education-related inequalities Most** ***versus*** **least educated**	**Difference in mortality per 1,000 live births (95% CI)**	**Odds Ratio of mortality (95% CI)**	**Difference in mortality per 1,000 live births (95% CI)**	**Odds Ratio of mortality (95% CI)**
Unadjusted	−3.9 (−5.8, −2.1)	0.77 (0.68, 0.87)	−9.7 (−11.6, −7.8)	0.43 (0.36, 0.51)
Adjusted for residence^a^	−3.2 (−4.9, −1.6)	0.81 (0.72, 0.91)	−8.5 (−10.4, −6.7)	0.48 (0.40, 0.56)
Adjusted for wealth rank	−3.0 (−4.5, −1.4)	0.83 (0.74, 0.93)	−6.9 (−8.8, −5.0)	0.55 (0.47, 0.65)
Adjusted for both covariates^c^	−2.5 (−4.1, −1.0)	0.85 (0.76, 0.95)	−6.4 (−8.2, −4.6)	0.58 (0.49, 0.68)

^a^Viet Nam was excluded from analyses on early neonatal mortality; all deaths happened among rural babies.

^b^The estimate of Viet Nam was adjusted for education only in analysis on early neonatal mortality.

^c^The estimate of Viet Nam was adjusted for wealth only in analysis on early neonatal mortality.

The slope index of inequality (SII) was used to estimate the mortality differences. Odds ratios are presented as the relative index of inequality (RII). Pooled estimates are from inverse-variance random-effects meta-analyses. Sample weights and robust standard errors were used in analyses.

95% CI = 95% Confidence Interval.

### Comparison of socioeconomic status and early neonatal mortality

**Figure 3:**
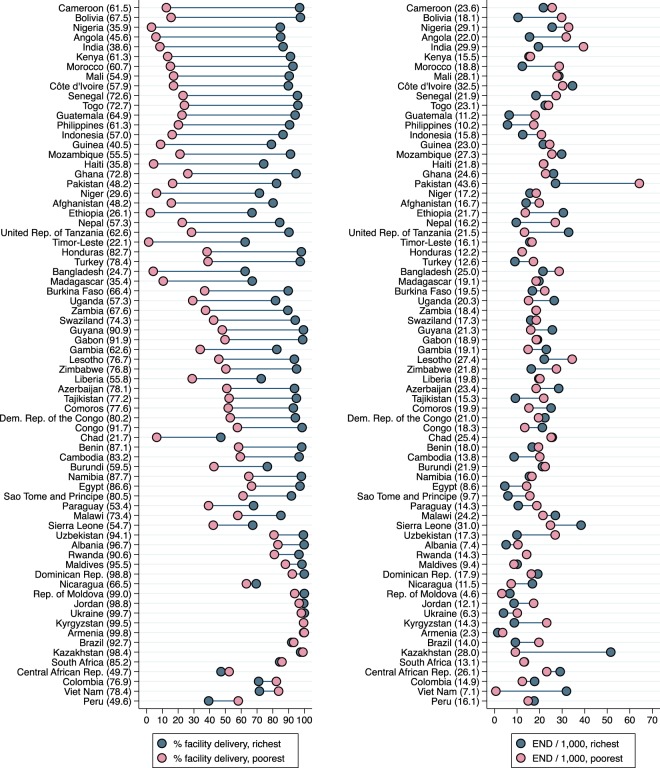
Unadjusted wealth-related inequalities in facility delivery and early neonatal mortality in 72 low- and middle-income Demographic and Health Survey countries. Country-level average prevalence of facility delivery (left) and early neonatal deaths per 1,000 live births (right) are shown in parentheses after the country name. Countries are sorted in descending order of inequality in facility delivery between the richest and poorest households. Countries with inverse inequalities i.e. *lower* percentage of facility deliveries among the richest compared with the poorest, such as Central African Republic, Colombia, Viet Nam and Peru, are at the bottom. Sample weights and robust standard errors were used in analyses. Pooled estimates are from inverse-variance random-effects meta-analyses. END/1,000 = early neonatal deaths per 1,000 live births. Graph command is from www.equidade.org.

**Figure 4:**
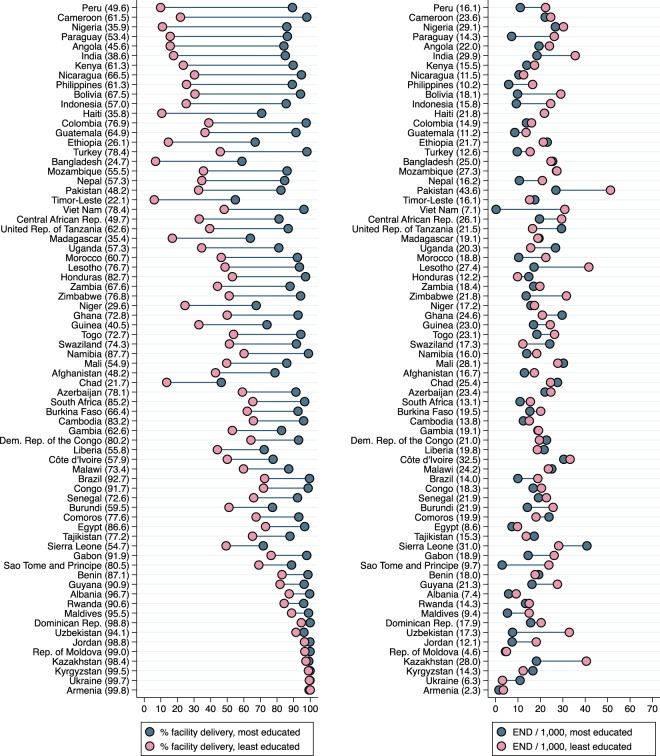
Unadjusted education-related inequalities in facility delivery and early neonatal mortality in 72 low- and middle-income Demographic and Health Survey countries. Country-level average prevalence of facility delivery (left) and early neonatal deaths per 1,000 live births (right) are shown in parentheses after the country name. Countries are sorted in descending order of inequality in facility delivery between the most and least educated mothers. Sample weights and robust standard errors were used in analyses. Pooled estimates are from inverse-variance random-effects meta-analyses. END/1,000 = early neonatal deaths per 1,000 live births. Graph command is from www.equidade.org.

In contrast to the small socio-economic inequalities in early neonatal mortality, differentials in facility delivery related to household wealth and maternal education were large in most countries (Figs 3 and 4).

In the unadjusted pooled analyses (Table 2), prevalence of facility delivery was 87% among the richest and 44% among the poorest translating into an absolute difference of 43%. For education, the absolute difference was 37%, as 51% and 88% of newborns belonging to the least and most educated mothers, respectively, were born in health facilities. After adjusting for the other two covariates, the inequalities related to wealth and education weakened, but remained considerable (28% and 22% difference; Odds Ratios 6.8 and 6.9, respectively). When using skilled birth attendant as an outcome instead of facility delivery, wealth-related inequalities were similar and education-related inequalities were slightly larger (Supplementary Table S2). In addition to individual-level analyses of facility delivery presented in Table 2, we studied inequalities in cluster-level facility delivery (which can be considered a measure of access to care, whereas individual-level facility delivery represents actual use of care). The associations of individual-level wealth and education with cluster-level facility delivery are presented in Supplementary Table S3.

**Table 2:** Individual-level wealth and education inequalities in facility delivery. Pooled unadjusted and adjusted estimates for 72 low- and middle-income Demographic and Health Survey countries. N = 667,478 deliveries.

Wealth-related inequalities Richest *versus* poorest	Percent difference in facility delivery (95% CI)	Odds Ratio of facility delivery (95% CI)
Unadjusted	42.9 (34.6, 51.2)	18.22 (12.99, 25.58)
Adjusted for residence	36.3 (28.8, 43.8)	11.96 (8.63, 16.57)
Adjusted for education rank	34.3 (28.6, 40.0)	10.36 (7.84, 13.70)
Adjusted for both covariates	27.5 (23.0, 31.9)	6.76 (5.25, 8.70)
**Education-related inequalities Most** ***versus*** **least educated**	**Percent difference in facility delivery (95% CI)**	**Odds Ratio of facility delivery (95% CI)**
Unadjusted	37.3 (31.0, 43.5)	20.10 (15.97, 25.30)
Adjusted for residence	29.8 (24.8, 34.7)	12.07 (9.78, 14.90)
Adjusted for wealth rank	24.3 (20.1, 28.6)	7.79 (6.38, 9.52)
Adjusted for both covariates	22.2 (18.9, 25.5)	6.85 (5.79, 8.10)

Sample weights and robust standard errors were used in analyses. Pooled estimates are from inverse-variance random-effects meta-analyses. The slope index of inequality (SII) was used to estimate the percent differences. Odds Ratios are presented as the relative index of inequality (RII).

95% CI = 95% Confidence Interval.

To avoid the issue of confounding by case-mix inherent to analyses of individual-level facility delivery and mortality, we analyzed the effect of average cluster-level facility delivery on mortality (Table 3). In unadjusted pooled analyses, we saw a small protective effect of cluster-level facility delivery: There were 4.1 deaths per 1,000 live births less in clusters with highest coverage of facility delivery than in clusters with lowest coverage (i.e. 21.6 per 1,000 vs. 17.5 per 1,000). When adjusting for residence, average wealth rank and mother’s education rank in the cluster, the difference reduced to 1.6 deaths per 1,000 live births and was not significant.

**Table 3:** Association between average proportion of facility delivery in the cluster and early neonatal mortality. Pooled unadjusted and adjusted estimates for 69^a^ low- and middle-income Demographic and Health Survey countries. N = 675,320 live births.

Clusters with highest *versus* lowest coverage of facility deliveries	Difference in mortality per 1,000 live births (95% CI)	Odds Ratio of mortality (95% CI)
Unadjusted	−4.1 (−6.1, −2.2)	0.77 (0.68, 0.87)
Adjusted for residence^b^	−3.6 (−5.4, −1.7)	0.81 (0.72, 0.91)
Adjusted for average wealth rank in cluster	−2.1 (−4.4, 0.0)	0.86 (0.74, 1.00)
Adjusted for average education rank in cluster	−2.3 (−4.3, −0.3)	0.87 (0.76, 0.99)
Adjusted for all of the above covariates^c^	−1.6 (−3.7, 0.5)	0.90 (0.78, 1.05)

^a^Armenia, Moldova and Ukraine were excluded from analyses; all deaths happened in clusters with 100% coverage of facility delivery in these countries.

^b^Viet Nam was excluded from analyses; all deaths happened among rural babies. ^c^Estimate for Viet Nam was adjusted for wealth and education only.

Sample weights and robust standard errors were used in analyses. Pooled estimates are from inverse-variance random-effects meta-analyses. The slope index of inequality (SII) was used to estimate the mortality differences. Odds Ratios are presented as the relative index of inequality (RII).

95% CI = 95% Confidence Interval.

For completeness, we also analyzed individual-level facility delivery and delivery assisted by skilled birth attendant and these were associated with a small reduction in early neonatal mortality in pooled crude analyses while there was no association when adjusted for residence, wealth or education with estimates close to the null value (Supplementary Tables S4 and S5).

## Discussion

Using individual-level data from 72 countries, we found that socioeconomic inequalities in early neonatal mortality were small, even in contexts with high overall mortality, in contrast to substantial inequalities in postneonatal infant mortality and in facility delivery. The inequalities related to education were slightly larger compared to wealth-related inequalities. Birth in a health facility was only slightly protective in individual- and cluster-level analyses. To our knowledge, this is the first study to report inequalities in early neonatal mortality on such a large scale and to compare them with inequalities in postneonatal infant mortality. The findings suggest that in many low- and middle-income settings, not even the socioeconomically advantaged are delivering in health facilities capable of providing high-quality care at birth.

These findings are consistent with the observation that neonatal mortality has declined only slowly during the past decades and remains high despite increasing coverage of facility delivery^29^, suggesting that quality of care at birth is not sufficiently high to save newborn lives^7,30,31^. Similar to our study, the DHS analysis from 67 low-and middle-income countries that included 1,473,226 live births found no association between individual-level facility delivery and early neonatal mortality^10^. They found a slight decrease in the odds of early neonatal mortality for predicted facility delivery (based on facility use for previous births), an analysis conducted to overcome confounding by case-mix^10^. A study on the Indian cash-incentive program Janani Suraksha Yojana reported an increase in utilization of maternity services especially among poorer and less educated women, however, whether the increased service use translated into reductions in neonatal or first-day mortality was not as clear^32^. The authors of both studies concluded that quality of care may have been inadequate to save lives. Our approach was to investigate quality of care at birth indirectly by comparing early newborn survival (which can be reduced with high quality of care, see Fig. 1) between the extremes of wealth and education distributions taking into account the entire population i.e. between socioeconomically advantaged mothers who have the means to seek the best available care and disadvantaged mothers who typically deliver at home without assistance and might only seek care in case of complications.

Higher education and wealth are known to be associated with increased use of skilled delivery care and better access to caesarean section^33–37^. Socioeconomic inequalities in skilled delivery attendance (that should reduce early neonatal mortality) are regularly larger than inequalities in interventions that reduce postneonatal infant mortality, which is unrelated to childbirth, such as immunization coverage, oral rehydration therapy or medical treatment for childhood illnesses, based on analyses of DHS data^34,38^. We intentionally left the in-between category of late neonatal mortality (death on day 7–27) out of our analyses as our aim was to compare inequalities in deaths related to childbirth with deaths that are not connected to childbirth. An analysis of DHS data from 48 low- and middle-income countries found strong wealth-related inequalities in use of antenatal care, caesarean section and facility delivery, but only the inequalities in antenatal care coverage were associated with inequalities in neonatal mortality in country-level meta-regression^37^. The strong socioeconomic inequalities in use of delivery care, consistently found in the literature and also shown in our study, are likely to reflect the generally better access to preventive and emergency care among the richest and the most educated^16^. That these did not sufficiently translate into improved newborn survival raises serious concerns about the quality of care at birth in DHS countries.

The Countdown initiative estimated that high coverage (>90%) of high-quality care at birth has the potential to save 77% of (total) neonatal deaths by 2020 in 75 high-burden countries^7^. Assuming that facility delivery can prevent 77% of early neonatal deaths, our pooled mortality inequalities (due to differences in coverage of delivery care alone) would amount to 6.6 per 1,000 live births for wealth and 5.8 per 1,000 live births for education. [For wealth calculated from: mortality among the poorest (19.9/1,000) x proportional difference in facility delivery between richest and poorest mothers (=0.429) × 0.77. For education calculated from: Mortality among the least educated (=20.3/1,000) x proportional difference in facility delivery between most and least educated mothers (=0.373) × 0.77]. These are larger inequalities than we found (2.9 per 1,000 for wealth and 3.9 per 1,000 for education).

It may be argued that the potential protective effect of facility delivery was lower during the time period the DHS data were collected (1990–2016) because new interventions, such as cord cleaning with chlorhexidine, have only become available more recently. On the other hand, a 77% reduction in early neonatal mortality might in fact underestimate the potential protective effect of facility delivery, as care at birth is likely to avert a higher proportion of early than of later neonatal deaths. Although the data were over a fairly large time span (up to 26 years apart), the majority of datasets (89%) were collected after the year 2000. Furthermore, quality of care and mortality usually change slowly. Indeed, the inequalities in early neonatal mortality would have been similar to what we reported in our main analyses (data between 1990 and 2016) if we had excluded data collected before 2000 (Supplementary Table S6) or before 2010 (Supplementary Table S7). Rather than studying the change in inequalities over time, our aim was to compare differences in inequalities between facility use and early neonatal mortality and between early neonatal and postneonatal infant mortality using the latest available dataset for each country.

Our analyses were controlled for distal determinants i.e. socioeconomic factors only as we wanted to capture the largest inequalities and include all mechanisms through which wealth and education can influence survival. As suggested by Mosley and Chen in their framework for child survival (1984), wealth and education are likely to improve newborn survival also through mechanisms other than facility delivery. These mechanisms include longer birth intervals, better antenatal care-seeking, better hygiene, better care-seeking for newborn sepsis, improved nutrition of the mother, and lower prevalence of small-for-gestational age babies^39^. Only few mechanisms would act in the other direction, e.g. maternal obesity, which is more common among the socioeconomically advantaged and can increase the risk of early neonatal death^40^. Given the higher use of delivery care among these populations, well-functioning health systems should be capable of mitigating these risks - which is the case in high-income settings where obesity is common and neonatal death is rare. Considering these multiple factors benefitting the richest and the most educated, the small inequalities identified are surprising and a cause for concern, as they indicate a lack of good childbirth care even for the most advantaged sub-population.

Given the cross-sectional nature of the DHS, the associations are not necessarily causal or unidirectional. A reverse effect of a newborn death on socioeconomic position is possible, e.g. through catastrophic health expenditure, particularly in poor households^39^. Wealth and education are also not necessarily comparable across countries and a rich household in one country might be a poor household in another. For this reason, we used cumulative wealth and education ranks which are relative measures within each country and studied mortality differences between the extremes of wealth and education distributions. Although the DHS follows a well-standardized protocol, it relies on retrospective reports by mothers, introducing potential error in mortality estimates, such as misclassification, heaping or underreporting of deaths^41,42^.

To study mortality related to childbirth, we would have ideally focused on deaths due to intrapartum-related causes (including stillbirths) that can be prevented by skilled delivery and perinatal care^3,7^. Our main analyses did not include stillbirths as the DHS only includes information on the latest stillbirth without information on place of delivery. Cross-tabulating the latest stillbirth by wealth quintile and education level showed that stillbirths were more commonly reported among the richest and the most educated (Supplementary Table S8). This means that including stillbirths to study perinatal mortality may have reduced inequalities even further. Due to lack of data on stillbirths and on cause of neonatal deaths, we investigated early neonatal mortality, of which a large proportion is related to complications at childbirth.

To investigate the role of facility delivery, we performed cluster-level analyses to overcome confounding by case-mix inherent to individual-level analyses. In these analyses we make the assumption that the prevalence of complications is similar across clusters. Even if this assumption does not hold, it is likely that socioeconomically disadvantaged clusters, which have lower facility delivery, have a higher prevalence of complications (e.g. due to poor access to antenatal care and poor nutrition) leading to higher early neonatal mortality. This would exaggerate any inequalities in mortality and cannot explain the small inequalities found. Furthermore, we accounted for this, at least in part, by adjusting the cluster-level analyses for cluster-level wealth and education.

Finally, early neonatal deaths are rare events even in contexts with high mortality, leading to large uncertainties in country-level estimates. Pooling analyses over 72 countries provided sufficient power to detect effects, and we did find strong inequalities for postneonatal infant mortality.

## Conclusions

The high levels of early neonatal mortality overall and in the majority of the countries studied highlight that early neonatal mortality remains a major global health problem that has not been solved by increasing coverage of facility deliveries. Socioeconomic inequalities in child mortality seem to increase with child age: inequalities are low in the early neonatal period, higher in the neonatal period^21^ and postneonatal infancy, and highest in under-five child mortality^22^. The socioeconomic inequalities in child mortality reported in the literature^2,17–22^ and the disparities shown for postneonatal infant mortality in this study demonstrate that a survival advantage often arises when better means of survival are available to some. The comparably small socioeconomic inequalities in early neonatal mortality in many high-mortality contexts imply that the survival benefit of being born to a rich and educated mother, most often in a health facility, was often very small. Quality of care may have been the broken link on the pathway between socioeconomic status and early neonatal survival (Fig. 1). Improvement in quality of care should thus be the priority in such settings, rather than increasing the proportion of facility deliveries further.

Finally, future studies should consider deaths related to childbirth as their own entity, separately from later infant mortality, as the biological mechanisms, socioeconomic determinants and thus the solutions for reducing these mortalities differ.

## Supplementary information

Supplementary information

## Data Availability

The data that support the findings of this study are available upon request from the Demographic and Health Surveys program or from the authors with permission of the Demographic and Health Surveys program.
