# Supplementary material for: Comparing socioeconomic inequalities between early neonatal mortality and facility delivery: Cross-sectional data from 72 low- and middle-income countries
Source: Sci Rep. 2019 Jul 5;9:9786. doi: 10.1038/s41598-019-45148-5 (PMC6611781; doi:10.1038/s41598-019-45148-5)
Supplement: Supplementary file 1 — Supplementary information [file 41598_2019_45148_MOESM1_ESM.pdf]

SUPPLEMENTARY INFORMATION

Comparing socioeconomic inequalities between early neonatal mortality and facility delivery: Cross-sectional data from 72 low- and middle-income countries

Terhi J. Lohela[1,2], M.D. Ph.D. (terhi.lohela@helsinki.fi)
Robin C. Nesbitt[2], Dr. sc. hum.
Juha Pekkanen[1,3], Ph.D.
Sabine Gabrysch[2], Ph.D.

[1] Department of Public Health, University of Helsinki, Helsinki, Finland
[2] Heidelberg Institute of Global Health, Heidelberg University, Heidelberg, Germany
[3] Environmental Health Unit, National Institute for Health and Welfare, Helsinki, Finland

Supplementary table S1. Unadjusted wealth-related and education-related inequalities in early neonatal mortality in 72 low- and middle-income Demographic and Health Survey countries.

Supplementary figure S1a. Unadjusted wealth-related inequalities in early neonatal mortality and postneonatal infant mortality in 72 low- and middle-income Demographic and Health Survey countries.

Supplementary figure S1b. Unadjusted education-related inequalities in early neonatal mortality and postneonatal infant mortality in 72 low- and middle-income Demographic and Health Survey countries.

Supplementary figure S2a. Unadjusted wealth-related inequalities in early neonatal mortality in 72 low- and middle-income Demographic and Health Survey countries using wealth quintiles.

Supplementary figure S2b. Unadjusted education-related inequalities in early neonatal mortality in 72 low- and middle-income Demographic and Health Survey countries using education level.

Supplementary table S2. Wealth-related and education-related inequalities in delivery assisted by a skilled birth attendant (SBA).

Supplementary table S3. Associations of individual-level wealth and individual-level education with cluster-level facility delivery.

Supplementary table S4. Association between facility delivery and early neonatal mortality.

Supplementary table S5. Association between care from skilled birth attendant and early neonatal mortality.

Supplementary table S6. Wealth-and education-related inequalities in early neonatal mortality in 64 low- and middle-income Demographic and Health Survey countries between 2000 and 2016.

Supplementary table S7. Wealth-and education-related inequalities in early neonatal mortality in 48 low- and middle-income Demographic and Health Survey countries between 2010 and 2016.

Supplementary table S8. Latest stillbirth within five years stratified by household wealth quintile and mother's level of education in 72 low- and middle-income Demographic and Health Survey countries.

Supplementary table S1. Unadjusted wealth-related and education-related inequalities in early neonatal mortality in 72 low- and middle-income Demographic and Health Survey countries.

			Wealth-related inequalities: Richest vs poorest		Education-related inequalities: Most vs least educated	
**Country (Year)**	**Live births[a]**	**Deaths per 1,000 live births[b]**	**Difference in mortality per 1,000 live births (95% CI) p-value**	**Odds Ratio of mortality (95% CI) p-value**	**Difference in mortality per 1,000 live births (95% CI) p-value**	**Odds Ratio of mortality (95% CI) p-value**
Afghanistan (2015)	32,686	16.7	-5.8 (-14.0, 2.3) p=0.1599	0.70 (0.42, 1.16) p=0.1666	-4.4 (-12.9, 4.0) p=0.3042	0.59 (0.19, 1.85) p=0.3643
Albania (2008)	1,616	7.4	-5.3 (-24.5, 14.0) p=0.5923	0.49 (0.04, 5.98) p=0.5768	-3.4 (-20.2, 13.4) p=0.6931	0.63 (0.07, 5.88) p=0.6839
Angola (2015)	14,322	22.0	-16.3 (-27.6, -5.0) p=0.0049	0.48 (0.29, 0.79) p=0.0039	-4.7 (-14.0, 4.7) p=0.3264	0.76 (0.44, 1.32) p=0.3333
Armenia (2015)	1,724	2.3	-2.1 (-6.7, 2.4) p=0.3607	0.41 (0.08, 1.97) p=0.2628	-2.0 (-6.7, 2.7) p=0.4070	0.43 (0.06, 2.84) p=0.3773
Azerbaijan (2006)	2,297	23.4	10.0 (-12.6, 32.7) p=0.3847	1.56 (0.59, 4.11) p=0.3680	-2.6 (-33.6, 28.4) p=0.8688	0.89 (0.23, 3.49) p=0.8682
Bangladesh (2011)	8,753	25.0	-7.2 (-20.3, 5.9) p=0.2805	0.74 (0.43, 1.28) p=0.2818	0.7 (-12.0, 13.5) p=0.9090	1.03 (0.58, 1.84) p=0.9088
Benin (2011)	13,380	18.0	-2.8 (-11.8, 6.2) p=0.5462	0.85 (0.51, 1.42) p=0.5443	1.6 (-5.8, 8.9) p=0.6727	1.15 (0.61, 2.16) p=0.6659
Bolivia (2008)	8,605	18.1	-19.4 (-33.1, -5.6) p=0.0059	0.34 (0.17, 0.70) p=0.0030	-19.3 (-33.7, -4.8) p=0.0090	0.31 (0.14, 0.71) p=0.0058
Brazil (1996)	5,044	14.0	-10.4 (-22.1, 1.3) p=0.0801	0.47 (0.20, 1.06) p=0.0671	-8.9 (-20.6, 2.8) p=0.1371	0.51 (0.21, 1.24) p=0.1355
Burkina Faso (2010)	15,033	19.5	-5.5 (-13.7, 2.7) p=0.1879	0.75 (0.49, 1.15) p=0.1873	-4.8 (-12.2, 2.6) p=0.2012	0.62 (0.28, 1.39) p=0.2439
Burundi (2010)	7,740	21.9	-1.2 (-15.4, 13.0) p=0.8669	0.94 (0.49, 1.84) p=0.8668	-11.5 (-20.7, -2.3) p=0.0148	0.45 (0.22, 0.90) p=0.0250
Cambodia (2014)	7,165	13.8	-11.4 (-25.4, 2.6) p=0.1090	0.43 (0.16, 1.16) p=0.0962	-2.6 (-15.1, 9.8) p=0.6790	0.81 (0.30, 2.20) p=0.6801
Cameroon (2011)	11,717	23.6	-3.9 (-16.0, 8.2) p=0.5291	0.84 (0.50, 1.43) p=0.5288	-2.5 (-12.6, 7.7) p=0.6323	0.88 (0.53, 1.48) p=0.6355
Central African Rep. (1994)	2,816	26.1	6.0 (-16.1, 28.0) p=0.5944	1.27 (0.53, 3.04) p=0.5976	-9.9 (-23.8, 4.1) p=0.1637	0.56 (0.24, 1.33) p=0.1901
Chad (2014)	18,600	25.4	0.5 (-11.1, 12.1) p=0.9336	1.02 (0.64, 1.63) p=0.9336	3.1 (-5.9, 12.1) p=0.4955	1.21 (0.71, 2.07) p=0.4773
Colombia (1995)	5,141	14.9	5.4 (-8.1, 19.0) p=0.4291	1.45 (0.59, 3.58) p=0.4193	-2.3 (-12.1, 7.6) p=0.6524	0.85 (0.43, 1.71) p=0.6531
Comoros (2012)	3,136	19.9	9.8 (-13.4, 33.1) p=0.4060	1.66 (0.50, 5.48) p=0.4027	5.8 (-13.7, 25.3) p=0.5575	1.45 (0.45, 4.70) p=0.5364
Congo (2011)	9,326	18.3	7.8 (-4.2, 19.8) p=0.2020	1.59 (0.79, 3.21) p=0.1920	-3.7 (-17.6, 10.2) p=0.5994	0.81 (0.36, 1.79) p=0.5364
Côte d'Ivoire (2011)	7,769	32.5	4.3 (-16.4, 25.1) p=0.6810	1.15 (0.59, 2.23) p=0.6808	-2.8 (-15.5, 9.9) p=0.6633	0.87 (0.46, 1.64) p=0.6689
Democratic Rep. of the Congo (2013)	18,714	21.0	2.8 (-6.9, 12.5) p=0.5696	1.15 (0.71, 1.85) p=0.5725	3.2 (-6.3, 12.7) p=0.5147	1.19 (0.71, 1.98) p=0.5119
Dominican Rep. (2013)	3,714	17.9	2.9 (-12.2, 17.9) p=0.7078	1.18 (0.49, 2.82) p=0.7112	-4.7 (-24.0, 14.7) p=0.6351	0.76 (0.25, 2.32) p=0.6326
Egypt (2014)	15,844	8.6	-9.7 (-16.1, -3.2) p=0.0034	0.32 (0.15, 0.66) p=0.0023	-2.4 (-8.9, 4.0) p=0.4551	0.73 (0.31, 1.72) p=0.4680
Ethiopia (2016)	10,641	21.7	16.9 (-5.6, 39.4) p=0.1415	2.27 (0.80, 6.45) p=0.1223	1.7 (-13.2, 16.6) p=0.8192	1.13 (0.42, 3.03) p=0.8147
Gabon (2012)	6,060	18.9	0.5 (-14.2, 15.2) p=0.9438	1.03 (0.46, 2.29) p=0.9439	-11.6 (-29.2, 6.0) p=0.1948	0.54 (0.22, 1.29) p=0.1651

Supplementary table S1. Unadjusted wealth-related and education-related inequalities in early neonatal mortality in 72 low- and middle-income Demographic and Health Survey countries. (continued)

			Wealth-related inequalities: Richest vs poorest		Education-related inequalities: Most vs least educated	
**Country (Year)**	**Live births[a]**	**Deaths per 1,000 live births[b]**	**Difference in mortality per 1,000 live births (95% CI) p-value**	**Odds Ratio of mortality (95% CI) p-value**	**Difference in mortality per 1,000 live births (95% CI) p-value**	**Odds Ratio of mortality (95% CI) p-value**
Gambia (2013)	8,086	19.1	8.1 (-4.8, 20.9) p=0.2168	1.56 (0.78, 3.09) p=0.2060	-0.3 (-12.0, 11.4) p=0.9595	0.98 (0.39, 2.46) p=0.9597
Ghana (2014)	5,884	24.6	3.5 (-14.4, 21.3) p=0.7014	1.16 (0.54, 2.47) p=0.7045	8.8 (-7.7, 25.3) p=0.2946	1.55 (0.69, 3.46) p=0.2840
Guatemala (2014)	12,440	11.2	-11.5 (-21.0, -2.0) p=0.0175	0.36 (0.16, 0.80) p=0.0120	-5.0 (-12.3, 2.2) p=0.1707	0.59 (0.28, 1.26) p=0.1733
Guinea (2012)	7,039	23.0	-2.8 (-18.9, 13.2) p=0.7286	0.88 (0.43, 1.80) p=0.7279	-7.4 (-18.0, 3.1) p=0.1641	0.54 (0.20, 1.44) p=0.2181
Guyana (2009)	2,165	21.3	9.6 (-16.4, 35.6) p=0.4681	1.61 (0.44, 5.89) p=0.4672	-11.4 (-36.9, 14.1) p=0.3812	0.57 (0.17, 1.90) p=0.3624
Haiti (2012)	7,245	21.8	0.1 (-12.9, 13.2) p=0.9851	1.01 (0.55, 1.85) 1.02 p=0.9851	0.1 (-13.4, 13.5) p=0.9922	1.00 (0.49, 2.07) p=0.9922
Honduras (2011)	10,887	12.2	-0.1 (-9.1, 9.0) p=0.9898	1.0 (0.47, 2.10) 2.0 p=0.9898	5.0 (-3.9, 13.9) p=0.2734	1.54 (0.73, 3.26) p=0.2583
India (2005)	51,547	29.9	-20.0 (-26.6, -13.4) p<0.0001	0.48 (0.38, 0.62) p<0.0001	-17.1 (-21.6, -12.5) p<0.0001	0.43 (0.33, 0.55) p<0.0001
Indonesia (2012)	18,009	15.8	-8.3 (-17.8, 1.2) p=0.0865	0.60 (0.33, 1.07) p=0.0840	-15.3 (-26.5, -4.2) p=0.0071	0.36 (0.18, 0.72) p=0.0036
Jordan (2012)	10,359	12.1	-8.7 (-26.1, 8.7) p=0.3277	0.49 (0.13, 1.88) p=0.3010	-10.8 (-21.3, -0.3) p=0.0446	0.40 (0.17, 0.95) p=0.0378
Kazakhstan (1999)	1,344	28.0	42.3 (10.7, 73.9) p=0.0089	6.70 (1.99, 22.54) p=0.0023	-22.3 (-72.5, 28.0) p=0.3835	0.44 (0.07, 2.77) p=0.3779
Kenya (2014)	20,964	15.5	-0.6 (-10.9, 9.7) p=0.9112	0.96 (0.49, 1.88) p=0.9113	-3.4 (-13.0, 6.2) p=0.4841	0.78 (0.38, 1.59) p=0.4931
Kyrgyz Rep. (2012)	4,363	14.3	-14.4 (-36.4, 7.7) p=0.2005	0.37 (0.09, 1.60) p=0.1841	4.4 (-12.5, 21.2) p=0.6110	1.36 (0.43, 4.33) p=0.6018
Lesotho (2014)	3,138	27.4	-12.4 (-34.2, 9.4) p=0.2650	0.63 (0.29, 1.40) p=0.2563	-24.5 (-51.7, 2.8) p=0.0785	0.40 (0.15, 1.05) p=0.0629
Liberia (2013)	7,597	19.8	-0.5 (-18.5, 17.5) p=0.9575	0.98 (0.39, 2.45) p=0.9575	3.0 (-8.6, 14.7) p=0.6102	1.22 (0.57, 2.61) p=0.6003
Madagascar (2008)	12,444	19.1	1.3 (-9.9, 12.4) p=0.8255	1.07 (0.59, 1.95) p=0.8256	0.5 (-8.9, 9.9) p=0.9167	1.03 (0.57, 1.86) p=0.9165
Malawi (2010)	19,967	24.2	5.5 (-4.9, 15.9) p=0.2971	1.26 (0.81, 1.96) p=0.2981	1.5 (-7.9, 10.9) p=0.7479	1.07 (0.70, 1.66) p=0.7468
Maldives (2009)	3,766	9.4	1.4 (-11.9, 14.7) p=0.8363	1.16 (0.28, 4.88) p=0.8353	-9.7 (-21.6, 2.2) p=0.1098	0.32 (0.08, 1.24) p=0.0993
Mali (2012)	10,311	28.1	0.8 (-12.4, 13.9) p=0.9067	1.03 (0.64, 1.66) p=0.9067	2.5 (-9.9, 14.8) p=0.6920	1.16 (0.56, 2.41) p=0.6852
Morocco (2003)	6,177	18.8	-16.4 (-30.7, -2.1) p=0.0251	0.42 (0.20, 0.87) p=0.0205	-12.1 (-19.7, -4.6) p=0.0017	0.31 (0.13, 0.73) p=0.0072
Mozambique (2011)	11,101	27.3	4.3 (-8.6, 17.3) p=0.5097	1.18 (0.73, 1.91) p=0.5101	-0.2 (-11.9, 11.6) p=0.9757	0.99 (0.58, 1.70) p=0.9757
Namibia (2013)	5,044	16.0	-1.1 (-15.8, 13.6) p=0.8812	0.93 (0.37, 2.36) p=0.8809	-4.5 (-17.9, 9.0) p=0.5145	0.74 (0.30, 1.82) p=0.5162
Nepal (2016)	5,038	16.2	-17.3 (-31.6, -3.0) p=0.0178	0.35 (0.16, 0.79) p=0.0112	-10.3 (-19.1, -1.4) p=0.0229	0.41 (0.18, 0.92) p=0.0312
Nicaragua (2001)	6,985	11.5	9.4 (-0.9, 19.8) p=0.0747	2.29 (0.98, 5.37) p=0.0555	-2.1 (-12.0, 7.8) p=0.6756	0.81 (0.29, 2.25) p=0.6810

Supplementary table S1. Unadjusted wealth-related and education-related inequalities in early neonatal mortality in 72 low- and middle-income Demographic and Health Survey countries. (continued)

			Wealth-related inequalities: Richest vs poorest		Education-related inequalities: Most vs least educated	
**Country (Year)**	**Live births[a]**	**Deaths per 1,000 live births[b]**	**Difference in mortality per 1,000 live births (95% CI) p-value**	**Odds Ratio of mortality (95% CI) p-value**	**Difference in mortality per 1,000 live births (95% CI) p-value**	**Odds Ratio of mortality (95% CI) p-value**
Niger (2012)	12,535	17.2	-2.8 (-13.2, 7.6) p=0.6011	0.85 (0.46, 1.58) p=0.6016	-1.6 (-9.8, 6.6) p=0.7056	0.85 (0.35, 2.06) p=0.7136
Nigeria (2013)	31,457	29.1	-7.3 (-15.3, 0.6) p=0.0716	0.77 (0.58, 1.02) p=0.0706	-3.6 (-10.2, 3.0) p=0.2858	0.84 (0.61, 1.16) p=0.2948
Pakistan (2012)	11,763	43.6	-37.2 (-57.1, -17.3) p=0.0003	0.41 (0.26, 0.64) p=0.0001	-24.4 (-35.6, -13.1) p<0.0001	0.39 (0.24, 0.63) p=0.0002
Paraguay (1990)	4,242	14.3	-8.3 (-21.2, 4.7) p=0.2102	0.55 (0.22, 1.40) p=0.2099	-19.0 (-31.4, -6.6) p=0.0029	0.26 (0.11, 0.62) p=0.0026
Peru (1996)	17,547	16.1	2.6 (-6.5, 11.8) p=0.5733	1.18 (0.66, 2.10) p=0.5755	-11.3 (-18.5, -4.1) p=0.0022	0.46 (0.28, 0.76) p=0.0024
Philippines (2013)	7,216	10.2	-11.7 (-21.2, -2.1) p=0.0165	0.33 (0.14, 0.77) p=0.0103	-10.7 (-20.9, -0.5) p=0.0402	0.35 (0.13, 0.91) p=0.0319
Rep. of Moldova (2005)	1,552	4.6	3.5 (-7.3, 14.3) p=0.5189	2.09 (0.25, 17.85) p=0.4979	-0.4 (-8.4, 7.6) p=0.9187	0.91 (0.16, 5.23) p=0.9191
Rwanda (2014)	7,850	14.3	0.0 (-10.2, 10.2) p=0.9928	1.0 (0.49, 2.07) 2.0 p=0.9928	-1.6 (-11.0, 7.7) p=0.7293	0.88 (0.42, 1.83) p=0.7304
Sao Tome and Principe (2008)	1,928	9.7	-9.7 (-26.0, 6.5) p=0.2367	0.38 (0.10, 1.47) p=0.1578	-20.9 (-55.6, 13.9) p=0.2357	0.11 (0.01, 2.49) p=0.1648
Senegal (2010)	12,326	21.9	-8.9 (-20.0, 2.1) p=0.1123	0.67 (0.40, 1.11) p=0.1166	-3.5 (-13.1, 6.1) p=0.4691	0.76 (0.34, 1.67) p=0.4916
Sierra Leone (2013)	11,915	31.0	13.6 (-0.5, 27.7) p=0.0590	1.57 (0.99, 2.49) p=0.0564	12.6 (-0.1, 25.3) p=0.0526	1.79 (1.05, 3.06) p=0.0326
South Africa (1998)	5,065	13.1	0.2 (-13.3, 13.6) p=0.9781	1.01 (0.36, 2.86) 1.02 p=0.9781	-4.6 (-21.2, 12.0) p=0.5856	0.69 (0.18, 2.68) p=0.5895
Swaziland (2006)	2,807	17.3	-2.5 (-22.5, 17.6) p=0.8090	0.87 (0.26, 2.83) p=0.8096	12.0 (-5.5, 29.5) p=0.1768	2.09 (0.76, 5.73) p=0.1525
Tajikistan (2012)	5,013	15.3	-12.6 (-28.6, 3.4) p=0.1235	0.42 (0.15, 1.15) p=0.0911	3.6 (-13.8, 20.9) p=0.6874	1.27 (0.41, 3.96) p=0.6816
Timor-Leste (2009)	9,805	16.1	-1.0 (-10.9, 8.9) p=0.8484	0.94 (0.50, 1.76) p=0.8490	2.3 (-7.4, 12.0) p=0.6421	1.19 (0.58, 2.43) p=0.6350
Togo (2013)	6,975	23.1	-1.4 (-14.7, 11.8) p=0.8312	0.94 (0.52, 1.68) p=0.8308	-7.9 (-20.0, 4.3) p=0.2032	0.62 (0.29, 1.34) p=0.2235
Turkey (2003)	4,533	12.6	-8.2 (-20.6, 4.2) p=0.1923	0.52 (0.19, 1.42) p=0.2018	-5.8 (-19.9, 8.4) p=0.4242	0.58 (0.14, 2.41) p=0.4502
Uganda (2011)	7,878	20.3	11.4 (-4.0, 26.8) p=0.1463	1.78 (0.81, 3.88) p=0.1490	11.1 (-2.7, 24.9) p=0.1154	1.83 (0.88, 3.83) p=0.1070
Ukraine (2007)	1,220	6.3	-6.1 (-24.3, 12.0) p=0.5079	0.39 (0.03, 4.97) p=0.4688	7.8 (-7.2, 22.9) p=0.3050	3.58 (0.33, 39.31) p=0.2956
United Rep. of Tanzania (2015)	10,233	21.5	19.6 (6.1, 33.1) p=0.0045	2.52 (1.38, 4.62) p=0.0028	12.9 (0.9, 25.0) p=0.0354	1.94 (1.09, 3.47) p=0.0250
Uzbekistan (1996)	1,324	17.3	-17.0 (-45.2, 11.3) p=0.2383	0.36 (0.08, 1.61) p=0.1804	-25.3 (-67.0, 16.3) p=0.2314	0.22 (0.02, 2.16) p=0.1923
Viet Nam (2002)	1,317	7.1	31.3 (-30.6, 93.2) p=	55.04 (2.11, 1435) p	-30.7 (-94.3, 32.9) p=0.3412	<0.01 (<0.01, 3.33) p=0.1088
Zambia (2013)	13,442	18.4	-0.1 (-11.4, 11.2) p=0.9845	0.99 (0.53, 1.86) p=0.9845	-2.8 (-12.7, 7.2) p=0.5878	0.85 (0.47, 1.54) 0.5896
Zimbabwe (2015)	6,132	21.8	-11.2 (-26.2, 3.9) p=0.1458	0.59 (0.28, 1.24) p=0.1629	-18.0 (-34.3, -1.6) p=0.0311	0.42 (0.19, 0.95) p=0.0368

[a]unweighted population count [b]weighted mortality. The Slope Index of Inequality (SII) was used to estimate the mortality differences. Odds ratios are presented as the relative index of inequality (RII). Pooled estimates are from inverse-variance random-effects meta-analyses. Sample weights and robust standard errors were used in analyses. 95% CI = 95% Confidence Interval.

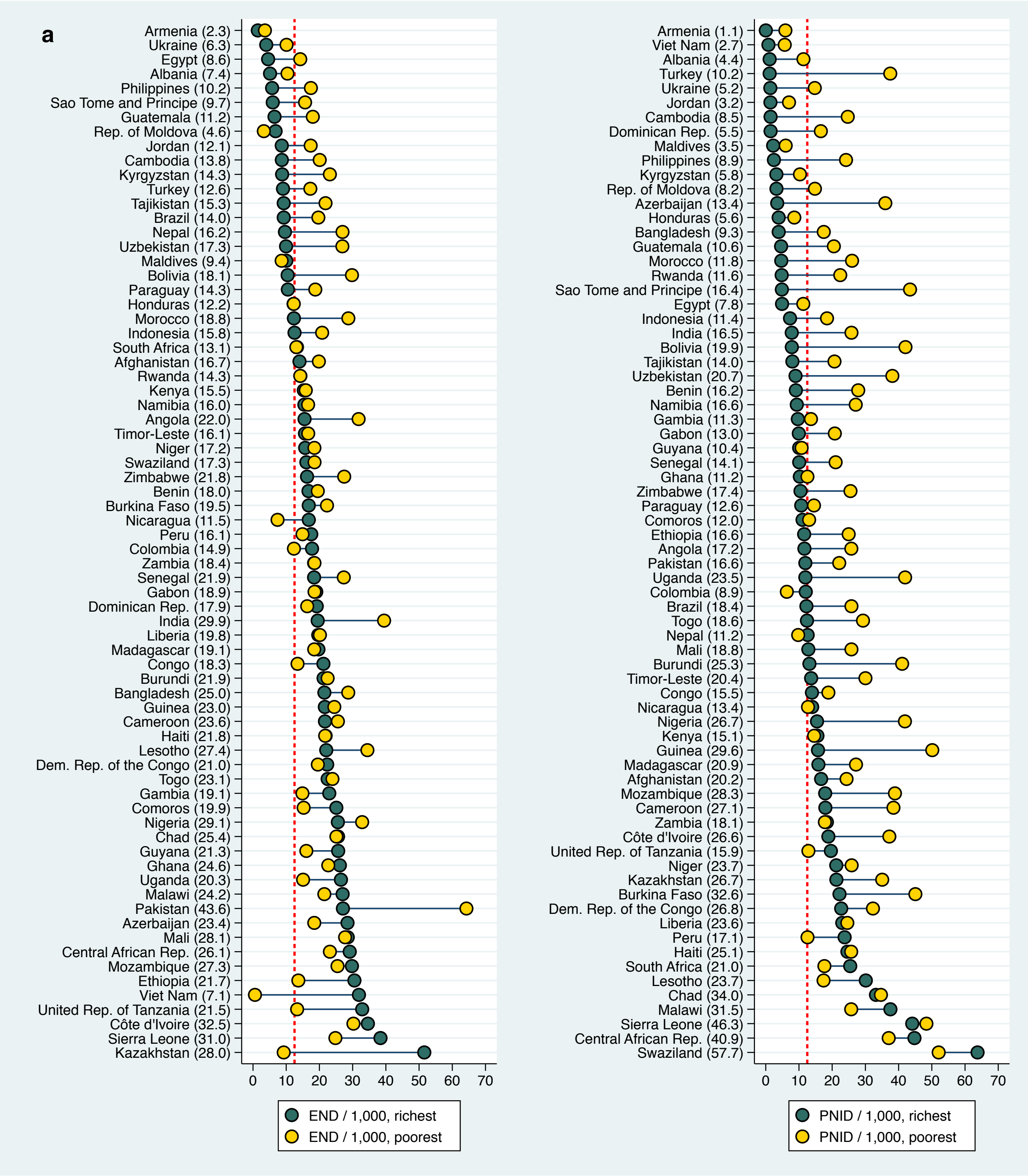

Supplementary figure S1a. Unadjusted wealth-related inequalities in early neonatal mortality and postneonatal infant mortality in 72 low- and middle-income Demographic and Health Survey countries. Country-level average mortality in each country is shown in parentheses. Countries are sorted in ascending order of mortality among the richest households. The graph shows a reference line for the Sustainable Development Goal (SDG) target of 12 neonatal deaths per 1,000 live births. Sample weights and robust standard errors were used in analyses. END / 1,000 = early neonatal deaths per 1,000 live births. PNID / 1,000 = postneonatal infant deaths per 1,000 live births. Graph command is from www.equidade.org.

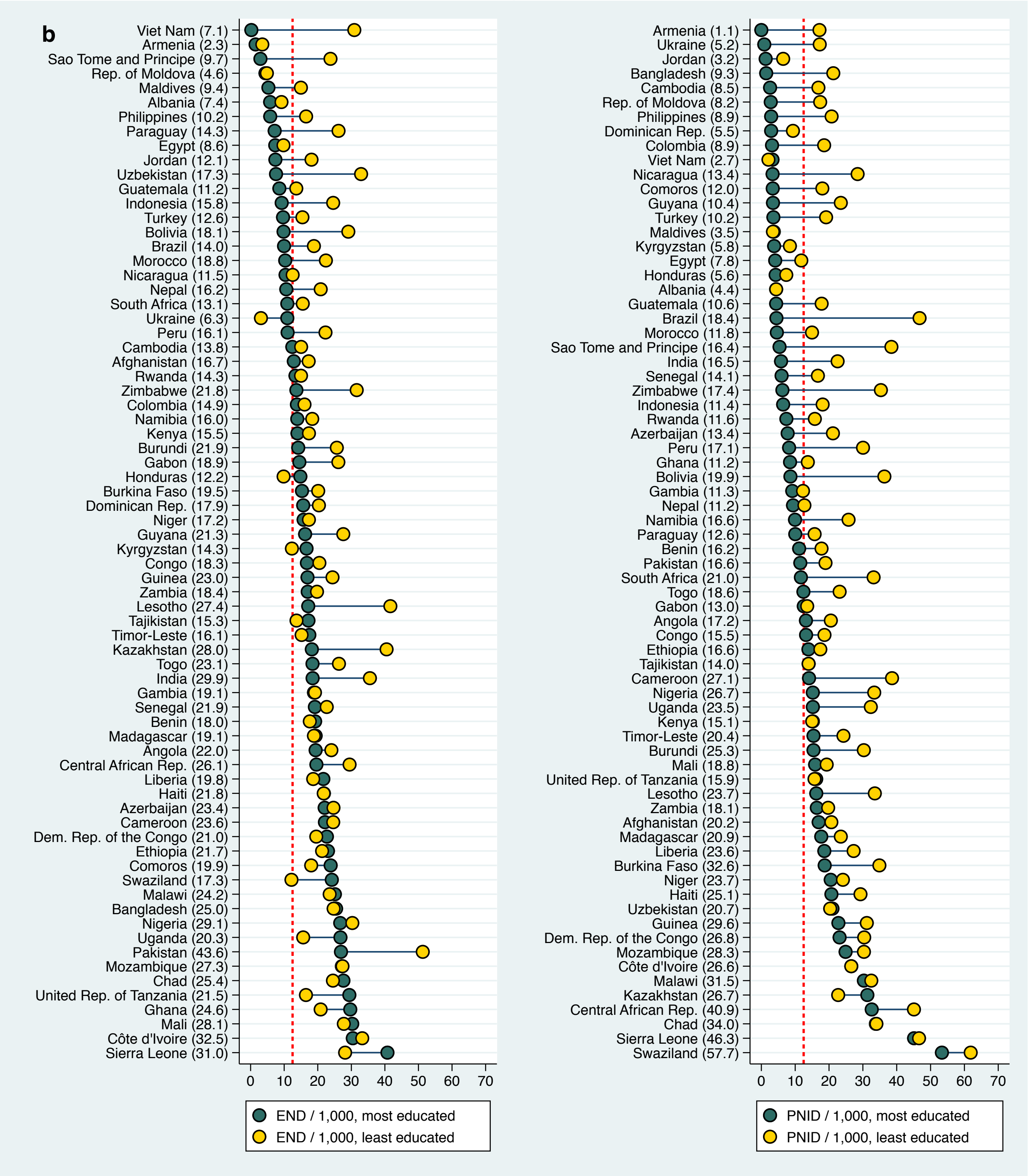

Supplemenatry figure S1b. Unadjusted education-related inequalities in early neonatal mortality and postneonatal infant mortality in 72 low- and middle-income Demographic and Health Survey countries. Country-level average mortality in each country is shown in parentheses. Countries are sorted in ascending order of mortality among the most educated mothers. The graph shows a reference line for the Sustainable Development Goal (SDG) target of 12 neonatal deaths per 1,000 live births. Sample weights and robust standard errors were used in analyses. END / 1,000 = early neonatal deaths per 1,000 live births. PNID / 1,000 = postneonatal infant deaths per 1,000 live births. Graph command is from www.equidade.org.

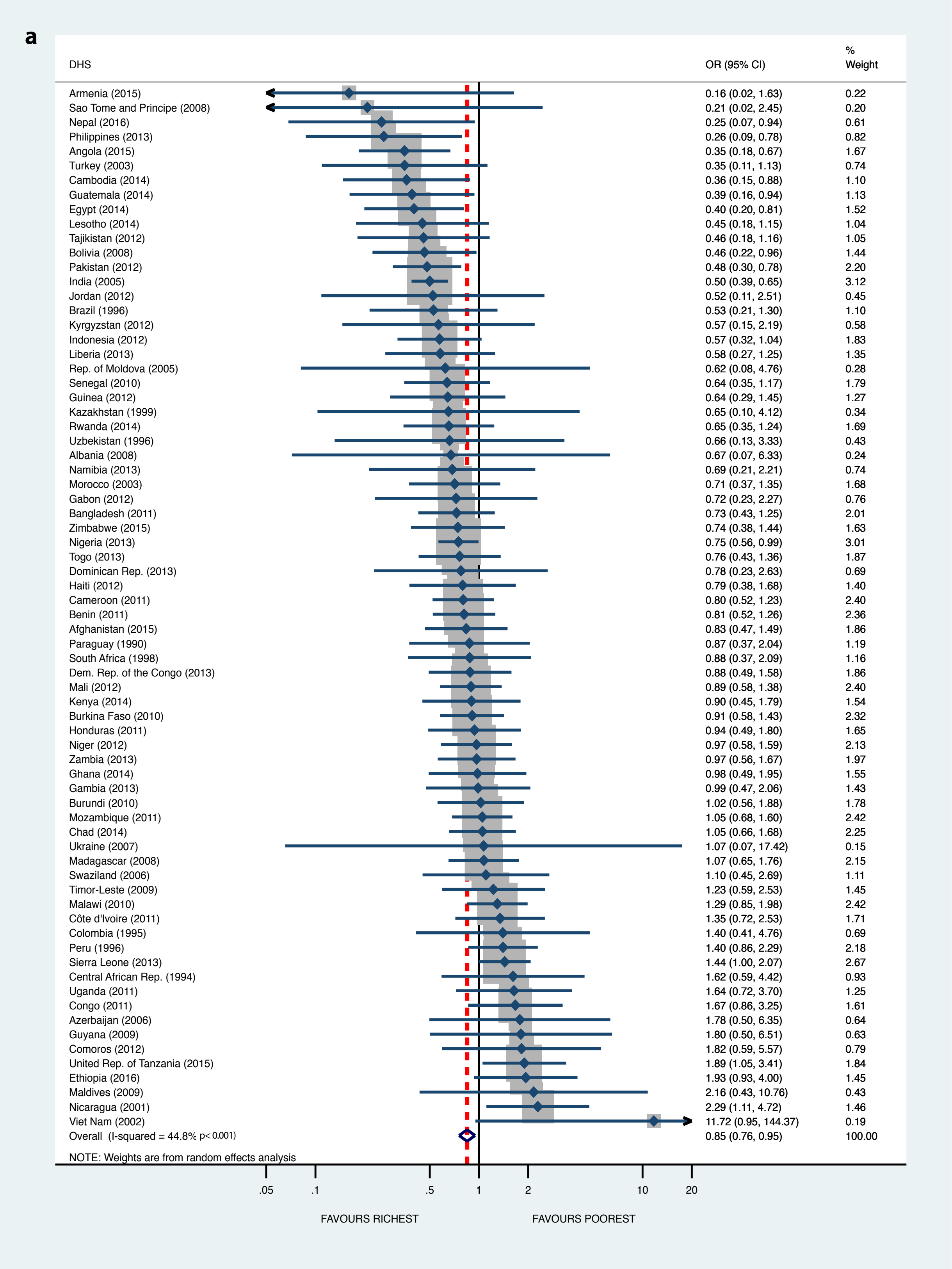

Supplementary figure S2a. Unadjusted wealth-related inequalities in early neonatal mortality in 72 low- and middle-income Demographic and Health Survey countries using wealth quintiles. In Brazil there were no deaths among the highest wealth quintile and in Armenia there were no deaths among the lowest quintile, and these quintiles were therefore merged with their adjacent group for the logistic regression analyses. Pooled overall estimates are from inverse-variance random-effects meta-analysis. Sample weights and robust standard errors were used in analyses. DHS = Demographic and Health Survey (Year). OR = Odds Ratio. 95% CI = 95% Confidence Interval.

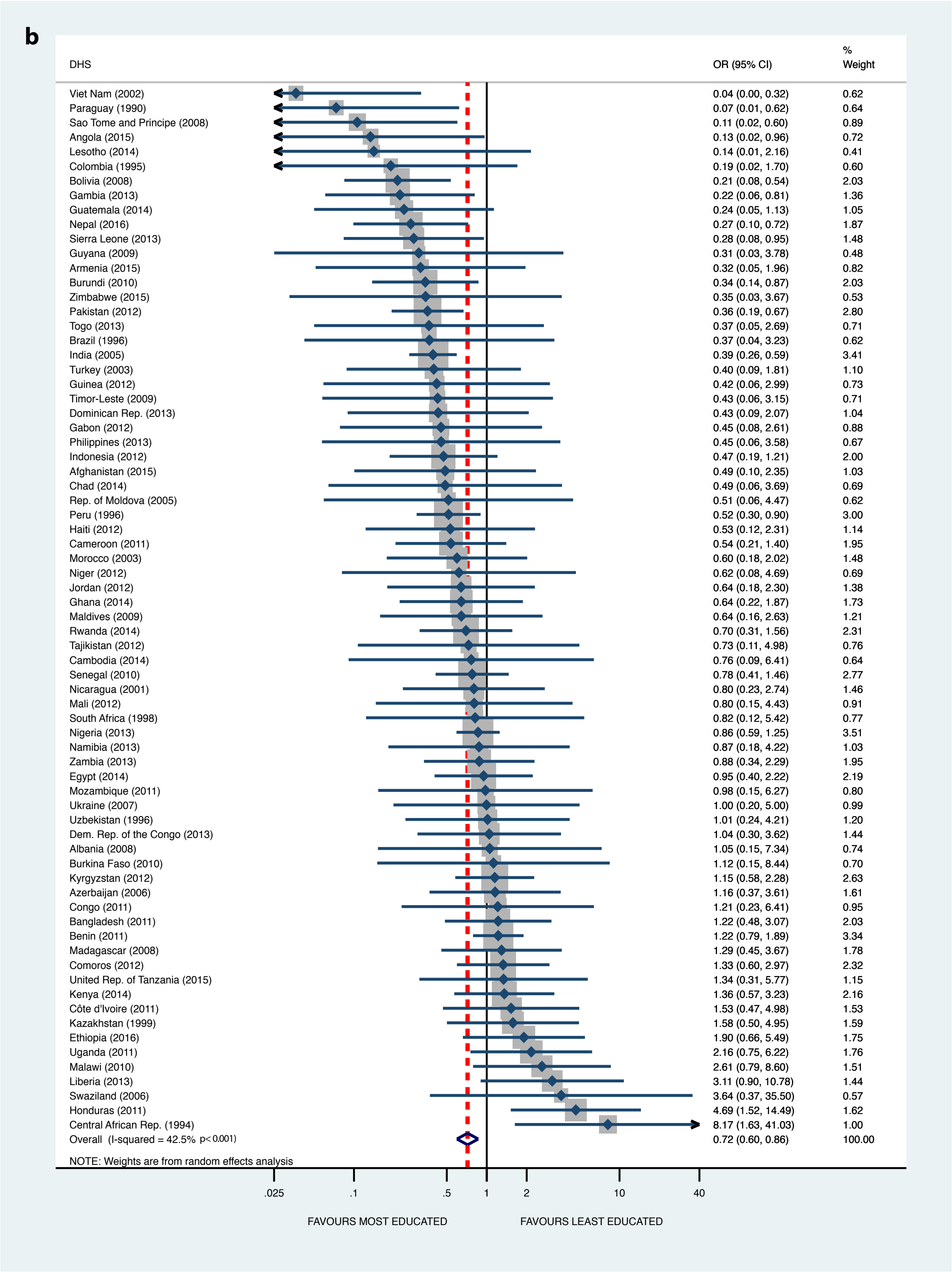

Supplementary figure S2b. Unadjusted education-related inequalities in early neonatal mortality in 72 low- and middle-income Demographic and Health Survey countries using education level. In 16 countries, there were no deaths among the highest or the lowest educational groups, and these education groups were therefore merged with their adjacent group for the logistic regression analyses. Pooled overall estimates are from inverse-variance random-effects meta-analysis. Sample weights and robust standard errors were used in analyses. DHS = Demographic and Health Survey (Year). OR = Odds Ratio. 95% CI = 95% Confidence Interval.

Supplementary table S2. Wealth-related and education-related inequalities in delivery assisted by a skilled birth attendant (SBA). Pooled unadjusted and adjusted estimates for 72 low- and middle-income Demographic and Health Survey countries. N=667,414 deliveries.

**WEALTH-RELATED INEQUALITIES richest *versus* poorest**	**Percent difference in SBA delivery (95% CI)**	**Odds Ratio of SBA delivery (95% CI)**
Unadjusted	42.5 (35.1, 49.9)	22.17 (15.58, 31.55)
Adjusted for residence	35.9 (29.5, 42.3)	14.14 (9.99, 20.01)
Adjusted for education rank	34.0 (28.7, 39.4)	12.55 (9.30, 16.93)
Adjusted for both covariates	27.3 (23.2, 31.3)	7.98 (6.04, 10.53)
**EDUCATION-RELATED INEQUALITIES most *versus* least educated**	**Percent difference in SBA delivery (95% CI)**	**Odds Ratio of SBA delivery (95% CI)**
Unadjusted	44.6 (38.5, 50.7)	20.93 (16.16, 27.13)
Adjusted for residence	35.5 (31.0, 40.0)	12.38 (9.74, 15.73)
Adjusted for wealth rank	28.8 (25.0, 32.7)	7.69 (6.13, 9.65)
Adjusted for both covariates	26.4 (23.1, 29.7)	6.81 (5.60, 8.29)

The Slope Index of Inequality (SII) was used to estimate the percent differences. Odds Ratios are presented as the relative index of inequality (RII). Pooled estimates are from inverse-variance random-effects meta-analyses. Sample weights and robust standard errors were used in analyses.
95% CI = 95% Confidence Interval.

Supplementary table S3. Associations of individual-level wealth and individual-level education with cluster-level facility delivery. Unadjusted and adjusted estimates for 72 low- and middle-income countries.

**Individual-level wealth**	**Cluster-level facility delivery Beta coefficient from linear regression (95% CI)**
Unadjusted	0.378 (0.324, 0.430)
Adjusted for residence	0.270 (0.245, 0.296)
Adjusted for education rank	0.328 (0.289, 0.368)
Adjusted for both covariates	0.225 (0.203, 0.248)
**Individual-level education**	**Cluster-level facility delivery Beta coefficient from linear regression (95% CI)**
Unadjusted	0.334 (0.293, 0.376)
Adjusted for residence	0.204 (0.181, 0.228)
Adjusted for wealth rank	0.160 (0.139, 0.180)
Adjusted for both covariates	0.122 (0.107, 0.137)

Pooled estimates are from inverse-variance random-effects meta-analyses. Sample weights and robust standard errors were used in analyses. 95% CI = confidence interval.

Supplementary table S4. Association between facility delivery and early neonatal mortality. Pooled unadjusted and adjusted estimates for 67[a] low- and middle-income Demographic and Health Survey countries. N=666,698 live births.

	**Difference in mortality per 1,000 live births (95% CI)**	**Odds Ratio of mortality (95% CI)**
Unadjusted	–1.4 (–2.9, 0.1)	0.90 (0.82, 0.98)
Adjusted for residence[b]	–0.5 (–2.1, 1.0)	0.95 (0.87, 1.03)
Adjusted for wealth rank	–0.3 (–2.1, 1.4)	0.94 (0.86, 1.04)
Adjusted for education rank	–0.3 (–1.8, 1.3)	0.96 (0.88, 1.05)
Adjusted for all of the above covariates	0.3 (–1.4, 2.1)	0.99 (0.90, 1.09)

[a]Albania, Armenia, Kyrgyzstan, Rep. of Moldova and Ukraine were excluded from analyses due to no deaths among those who were born outside of health facilities. The majority of births took place in a health facility in these countries.
[b]Viet Nam was excluded from analyses: all deaths happened among rural babies.
The Slope Index of Inequality (SII) was used to estimate the mortality differences. Odds Ratios are presented as the relative index of inequality (RII). Pooled estimates are from inverse-variance random-effects meta-analyses. Sample weights and robust standard errors were used in analyses.
95% CI = 95% Confidence Interval.

Supplementary table S5. Association between care from skilled birth attendant and early neonatal mortality. Pooled unadjusted and adjusted estimates for 65[a] low- and middle-income Demographic and Health Survey countries. N=663,978 live births.

	**Difference in mortality per 1,000 live births (95% CI)**	**Odds Ratio of mortality (95% CI)**
Unadjusted	–1.4 (–3.0, 0.2)	0.90 (0.82, 0.98)
Adjusted for residence[b]	–0.5 (–2.1, 1.1)	0.95 (0.87, 1.03)
Adjusted for wealth rank	–0.3 (–2.0, 1.5)	0.95 (0.86, 1.04)
Adjusted for education rank	–0.2 (–1.8, 1.4)	0.96 (0.88, 1.05)
Adjusted for all of the above covariates	0.4 (–1.3, 2.1)	0.99 (0.90, 1.09)

[a]Albania, Armenia, Kazakhstan, Kyrgyzstan, Rep. of Moldova, Ukraine and Uzbekistan were excluded from analyses due to no deaths among those without skilled attendant at birth. The majority of births were assisted by a skilled birth attendant in these countries.
[b]Viet Nam was excluded from analyses: all deaths happened among rural babies.
The Slope Index of Inequality (SII) was used to estimate the mortality differences. Odds Ratios are presented as the relative index of inequality (RII). Pooled estimates are from inverse-variance random-effects meta-analyses. Sample weights and robust standard errors were used in analyses.
95% CI = 95% Confidence Interval.

Supplementary table S6. Wealth-related and education-related inequalities in early neonatal mortality in 64 low- and middle-income Demographic and Health Survey countries between 2000 and 2016.		
**WEALTH- INEQUALITIES Richest *versus* poorest**	**Difference in mortality per 1,000 live births (95% CI)**	**Odds Ratio of mortality (95%)**
Unadjusted	-3.2 (-5.4, -1.0)	0.84 (0.74, 0.96)
Adjusted for education rank and residence[a]	-1.9 (-3.8, 0.0)	0.92 (0.82, 1.03)
**EDUCATION-INEQUALITIES Most *versus* least educated**	**Difference in mortality per 1,000 live births (95% CI)**	**Odds Ratio of mortality (95%)**
Unadjusted	-3.3 (-5.3, -1.4)	0.80 (0.70, 0.92)
Adjusted for wealth rank and residence[b]	-2.1 (-3.7, -0.5)	0.88 (0.79, 0.99)

[a]The estimate of Viet Nam was adjusted for education only in analysis on early neonatal mortality (all deaths happened among rural babies).

[b]The estimate of Viet Nam was adjusted for wealth only in analysis on early neonatal mortality (all deaths happened among rural babies).

The slope index of inequality (SII) was used to estimate the mortality differences. Odds ratios are presented as the relative index of inequality (RII). Pooled estimates are from inverse-variance random-effects meta-analyses. Sample weights and robust standard errors were used in analyses. 95% CI = confidence interval.

Supplementary table S7. Wealth-related and education-related inequalities in early neonatal mortality in 48 low- and middle-income Demographic and Health Survey countries between 2010 and 2016.		
**WEALTH- INEQUALITIES Richest *versus* poorest**	**Difference in mortality per 1,000 live births (95% CI)**	**Odds Ratio of mortality (95%)**
Unadjusted	-2.8 (-5.1, -0.6)	0.86 (0.75, 0.97)
Adjusted for education rank and residence	-2.1 (-4.2, 0.0)	0.92 (0.81, 1.04)
**EDUCATION-INEQUALITIES Most *versus* least educated**	**Difference in mortality per 1,000 live births (95% CI)**	**Odds Ratio of mortality (95%)**
Unadjusted	-2.7 (-4.6, -0.9)	0.85 (0.74, 0.96)
Adjusted for wealth rank and residence	-1.2 (-2.9, 0.4)	0.94 (0.84, 1.05)

The slope index of inequality (SII) was used to estimate the mortality differences. Odds ratios are presented as the relative index of inequality (RII). Pooled estimates are from inverse-variance random-effects meta-analyses. Sample weights and robust standard errors were used in analyses. 95% CI = confidence interval.

Supplementary table S8. Latest stillbirth within five years stratified by household wealth quintile and mother's level of education in 72 low- and middle-income Demographic and Health Survey countries. N=697,329 births.

	**Live births[a]** **N=679,818**	**Stillbirths[a] (per 1,000 births) N=17,511**	**Weighted stillbirths[b] per 1,000 births**
**Wealth quintile**			
Poorest (Q1)	168,092	3,723 (21.7)	22.5
Poorer (Q2)	146,922	3,507 (23.3)	24.0
Middle (Q3)	135,386	3,547 (25.5)	26.5
Richer (Q4)	123,291	3,355 (26.5)	27.3
Richest (Q5)	106,127	3,379 (30.9)	31.6
**Education level**			
None	244,321	4,867 (19.5)	20.3
Primary	211,246	4,967 (23.0)	23.9
Secondary/higher	224,251	7,677 (33.1)	34.1

[a]Unweighted population count
[b]Sample weights and robust standard errors were used.
Stillbirth is defined as a third trimester stillbirth i.e. death of a baby later than six months of gestation. Only the most recent stillbirth within the last five years is considered, not multiple ones per woman.
